# On the Dissection of Living Animals

**Published:** 1816-12

**Authors:** 


### *For the London Medical and Physical Journal.*

### *On the Dissection of Living Animals;* by AMICUS.

YOU will receive this from a constant reader, one who sincerely wishes success to your useful Journal, and whose contributions to it you have more than once honoured by publication. The manner in which you have, by several papers, joined the popular cry against experiments on living animals has excited a smile at your expence. It is possible to be humane without sacrificing the interests of science. It would have been better, perhaps, had you followed the example of other medical journalists, and said nothing on the subject. As you have so often introduced it, I would suggest to you to give as an extract the whole of a paper entitled, "On Cruelty to Animals," signed Philanthropos, in the New Monthly Magazine for Sept. last. You will thus, without at all lessening your claims to humanity, explain your sentiments respecting experiments on living animals in the way in which I am persuaded you wish them to be understood.

---

[We are much obliged by the above friendly hint; but, in defending ourselves, we do not mean to avail ourselves of the aid of any other Journal, especially one directed to the public at large, who are incompetent judges on these subjects. If we are accused of objecting to operations on living animals, in illustration of any point which may be serviceable to man, we will either explain, or join in the laugh at our own prudery. We, therefore, have only to request those who laugh at us to speak out, that we may correct our errors, or defend our positions.—EDIT.]

